# Comparative Anatomical Analyses of the Forearm Muscles of *Cebus libidinosus* (Rylands *et al.* 2000): Manipulatory Behavior and Tool Use

**DOI:** 10.1371/journal.pone.0022165

**Published:** 2011-07-15

**Authors:** Tales Alexandre Aversi-Ferreira, Rafael Souto Maior, Frederico O. Carneiro-e-Silva, Roqueline A. G. M. F. Aversi-Ferreira, Maria Clotilde Tavares, Hisao Nishijo, Carlos Tomaz

**Affiliations:** 1 Department and Faculty of Nursing, Neurosciences and Primates Behavior Center (NECOP), Federal University of Goiás, Catalão, Brazil; 2 Department of Physiology, School of Medicine and Pharmaceutical Sciences, System Emotional Science, University of Toyama, Toyama, Japan; 3 Department of Physiology, Institute of Biology, Laboratory of Neuroscience and Behavior, University of Brasilia, Brasilia, Brazil; 4 Department of Animal Anatomy, Faculty of Veterinary Medicine, Federal University of Uberlândia, Uberlândia, Brazil; Texas A&M University, United States of America

## Abstract

The present study describes the flexor and extensor muscles in *Cebus libidinosus'* forearm and compares them with those from humans, chimpanzees and baboons. The data is presented in quantitative anatomical indices for similarity. The capuchin forearm muscles showed important similarities with chimpanzees and humans, particularly those that act on thumb motion and allow certain degree of independence from other hand structures, even though their configuration does not enable a true opposable thumb. The characteristics of *Cebus'* forearm muscles corroborate the evolutionary convergence towards an adaptive behavior (tool use) between *Cebus* genus and apes.

## Introduction

In the last two decades, several behavioral studies have focused on the capuchin's ability to use tools. In its strictest sense, tool use is only found in a handful of old world monkeys (OWM) and apes. The only exceptions among new world monkeys (NWM) are the capuchins, which have been reported to use tools both in the captivity and in the wild [1], [2], [3]. Such studies have reported that the *Cebus* is capable to handle rocks to open coconuts, to use toothpicks to push food out of a pipe or to extract molasses through the orifices of a box [4], [5], [6]. Recently wild capuchins were observed to fish for termites using twigs, an activity until then only seen in chimpanzees [7]. Such complex behaviors are dependent on versatile grasping ability [8], [9]. Accordingly, *Cebus* have been reported to display a wide array of grasping strategies and manipulative, comparable to chimpanzees and humans [10], [11].

Dexterous hand ability, and consequently tool use, is associated with the development of primate intelligence and culture [12], [13]. This adaptive behavior therefore denotes an important evolutionary convergence, especially between capuchins and chimpanzees. Capuchin tool use seems also dependent on other neurological, cognitive and morphological convergences [10], [14]. In this sense, capuchins stand as an important model for testing hypotheses regarding the evolution of primate cognition.

Comparative anatomical analysis of primates may yield important knowledge regarding behavior and phylogeny. More specifically, forearm anatomy is crucial to understand manipulative behaviors of the hand. Although a few studies have focused on comparative behavioral assessment of capuchin tool use [8], [9], the literature on their forearm myology is scarce. Early studies have indicated that precision grips were untenable to capuchins due to lack of saddle joint in the hand and therefore tool use ability was not related to thumb mobility [15], [16]. Further behavioral studies, however, have reported that this genus can adduct the thumb towards the index finger, favoring the flexing of the interphalangeal rather than the metacarpophalangeal joint, coined ‘lateral opposability’ [8]. However, there are still no anatomical confirmations of these findings.

In the present study, the flexors muscles of the forearm in the *Cebus libidinosus* [17] monkey were investigated. Origin, insertion, arterial branching and innervation of each muscle were characterized to provide an anatomical understanding of the manual skills observed in *Cebus*. The anatomical observations here were then compared to the analogous muscles found in humans [18] and chimpanzees and baboons [19]. The degree of anatomical similarity among the forearm muscles in these species was compared using the Comparative Anatomy Index (CAI) [20].

## Materials and Methods

### Samples

Eight adult capuchin specimens (*Cebus libidinosus*) were used (seven males and one female) weighing from one to three kilograms. No animal was killed for the purposes of this study: four of them suffered accidental deaths in their natural habitats and were acquired from anatomical collection of the Neuroscience and Behavior of Primates Laboratory (NECOP) from the Federal University of Goias- Catalão-Goias. The remaining of them belonged to the Brazilian Institute of Environment and Renewable Natural Resources (IBAMA) archive and were donated to the University of Goiás in the 1970's. This work was approved by the Institutional Ethical Committee from the Federal University of Goiás (CoEP-UFG 81/2008, authorization from the IBAMA number 15275).

### Preparation of the animals for dissection

All procedures involving the animals were done in accordance to the guidelines of the Brazilian Society of Animal Experimentation (COBEA). After the trichotomy with a razor blade, the animals were incubated in water at room temperature for 10–12 hours; and then received perfusion, by the femoral vein, 10% of formaldehyde with 5% of glycerin for fixation. The animals were conserved in 10% of formaldehyde, in covered opaque cubes, to avoid the penetration of light and the evaporation of the formaldehyde.

To undertake the anatomical observations for the present work, after receiving the specimens at the anatomical collection of the Federal University of Goiás each one was processed, by the first author, as follows: (1) it received an injection of latex 601-A (Dupont) stained with Wandalar red diluted in ammonium hydroxide in the abdominal aorta in order to facilitate the visualization of small arteries; (2) it was incubated in water at room temperature for 10–12 hr; and then (3) it received a perfusion of 10% formaldehyde with 5% glycerin through the femoral vein for fixation. The monkeys were preserved in 10% formaldehyde in closed opaque boxes to avoid light penetration and formaldehyde evaporation.

### Dissection and documentation

The dissection of the forearm was performed with emphasis on the flexors muscles of the forearm and registered with a digital camera. The first author of the present paper dissected both sides of the eight specimens of *Cebus libidinosus*. The nomenclature of the forelimb muscles follows, whenever it is possible, that used in human anatomy (The Federative Committee on Anatomical Terminology, 1998). When no such parallel was possible, they were referred to following the patterns of the international nomenclature from the Human Anatomic Nominal. The data collected were analyzed and compared with the patterns described for human, chimpanzee and baboon species.

### Statistical analysis

Based on Aversi-Ferreira [20], we used a simple comparative non-parametric method for two different species associated on anatomical concepts of normality and variation as nominal variables. Relative frequency (**RF**) was defined as: **RF = (N−nv)/N**; where **N** is the total number of specimens of the sample and **nv** is the number of individuals presenting variation of the normal pattern.

When more than one parameter (location, nerve, blood vessel, origin and insertion of a muscle) was necessary, they were associated to a specific pondered value with respect to their degree of relevance in comparative analysis. Parameters with less variation were ascribed a higher value. Therefore, innervation, origin and insertion, and vascularization were ascribed the weighs 3, 2, 1, respectively.

The Pondered Average of Frequencies (**PAF**) was calculated using the RF values:


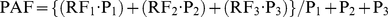
 where **RF_1_** is the frequency of the muscle innervation and **P_1_** is 3; **RF_2_** is the frequency of the muscle origin and **P_2_** is 2, **RF_3_** is the frequency of the muscle vascularization and **P_3_** is 1.

To consider the proximity between the structures studied, the difference in the relative frequency is calculated, or Comparative Anatomy Index (**CAI**) between samples from different species:


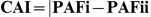
; where indexes i and ii represent samples 1 and 2.

From the equations above, it follows that the close to zero **CAI** values represents greater similarity between samples it represents, whereas a **CAI** closer to 1.0 means higher divergence between samples. More specifically, **CAI** value of 0 indicates **high similarity** between the structures analyzed, from 0 to 0.200 as **similar** structures, from 0.200 to 0.650 as **somewhat similar**, from 0,650 and 1,000 as **dissimilar**.

For the purposes of the present work, the *Cebus* was primarily chosen as the reference for comparison against human, chimpanzee and baboon morphology, although they were also analyzed among themselves.

For example, regarding the muscle flexor carpi radialis, **RF** was 1 to all parameters in *Cebus* specimens and **RF** = 0 was set for the absence of any parameters in the other species.

Then,
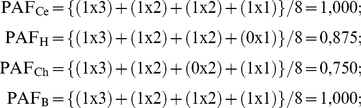



Where the indexes Ce, H, Ch and B represents respectively *Cebus*, humans, chimpanzees and baboons. In order, the first parameter is innervation, the second is origin of muscle, the third is insertion of muscle, and fourth is vascularization (see table 1). Note that vascularization is the only difference between *Cebus* and humans whereas insertion differs between *Cebus* and chimpanzees and no differences were observed between *Cebus* and baboons in this muscle.

**Table 1:** Comparative analyses of the flexor superficial muscles forearm among *Cebus libidinosus* (*C.l.*), human (*Homo*), chimpanzee (*Pan*) and baboon (*Papio*).

Muscle	Features	*Cebus libidinosus*	*Homo*	*Pan*	*Papio*
**Flexor carpi ulnaris**	**Origin**	Medial epicondyle of humerus and olecranon	Highly similar to *C.l.* **CAI = 0.0**	Highly similar to *C.l.* **CAI = 0.0**	Highly similar to *C.l.* **CAI = 0.0**
	**Insertion**	Pisiform bone			
	**Innervation**	Ulnar nerve			
	**Vascularization**	Ulnar artery			
**Palmaris longus**	**Origin**	Medial epicondyle of humerus	Variable, may be absentSomewhat similar to *C.l.* **CAI = 0.425**	Highly similar to *C.l.* **CAI = 0.0**	Highly similar to *C.l.* **CAI = 0,0**
	**Insertion**	Palmar aponeurosis			
	**Innervation**	Median nerve			
	**Vascularization**	Ulnar artery			
**Flexor carpi radialis**	**Origin**	Medial epicondyle of humerus	Similar to *C.l.*Vascularized by radial artery**CAI = 0.125**	Double insertion in metacarpal II and IIISomewhat similar to *C.l.* **CAI = 0.250**	Highly similar to *C.l.* **CAI = 0.0**
	**Insertion**	Base of metacarpal II			
	**Innervation**	Median nerve			
	**Vascularization**	Ulnar artery			
**Flexor digitorum superficialis**	**Origin**	Humeral head – medial epicondyle of the humerus; Radial head – anterior surface of the radius	Three heads of origin – humeral, radial and ulnar.Somewhat similar to *C.l.* **CAI = 0.375**	Highly similar to *Homo*Somewhat similar to *C.l.* **CAI = 0.375**	Highly Similar to *C.l.* **CAI = 0.0**
	**Insertion**	Middle phalanges of II to V fingers			
	**Innervation**	Median nerve			
	**Vascularization**	Ulnar artery and branches of the radial artery			
**Pronator teres**	**Origin**	Medial epicondyle of humerus	Two heads of origin, humeral and ulnarSomewhat similar to *C.l.* **CAI = 0.375**	Highly similar to *Homo*Somewhat similar to *C.l.* **CAI = 0.375**	Highly similar to *C.l.* **CAI = 0.0**
	**Insertion**	Postero-lateral portion of the radius			
	**Innervation**	Median nerve			
	**Vascularization**	Ulnar artery			
**Superficial group**			Somewhat similar**GCAI = 0.26**	Similar **GCAI = 0.20**	Highly similar **GCAI = 0.0**

From these values, the CAI is calculated,
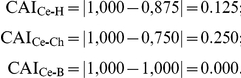



The CAI calculated indicate that the muscle flexor carpi radialis of *Cebus* and baboons are **highly similar**, *Cebus* and humans are **similar**, and *Cebus* and chimpanzees are **somewhat similar** structures, according to the parameters adopted here.

A special case is the palmaris longus muscle that is absent in 10% of human population [18] and that presents many variations. To calculate the **FR**, the same purposed pondered values were used, but they were adjusted by a 10% decrease to innervation and vascularization. Adjustment to origin and insertion were set at 50% decrease since Gray [18] observed that variations in this muscle occur mainly found at its origin and insertion.

Other important parameter to be calculated is the ‘**Group CAI**’ (**GCAI**) for structures, such as superficial and deep muscles in the forearm, which combines the summation **RF** of individual muscles summation average of **CAI** for the species not used as reference (*Homo*, *Pan* and *Papio*). To calculated **GCAI** we used the following equation:
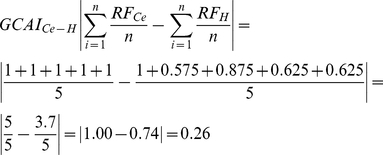
or,
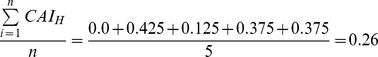


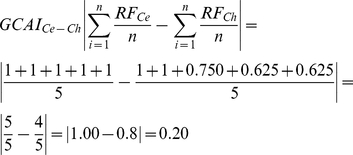
or,
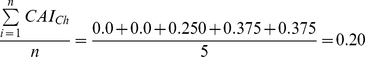


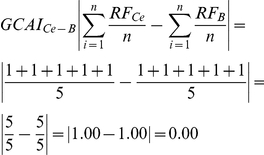
or,
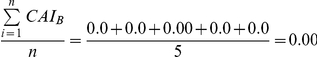



The GCAI calculated above shows that the group of superficial muscles of *Cebus*'s forearm is highly similar to baboon, similar to chimpanzees, and somewhat similar to humans.

## Results and Discussion

Recently, we have reported a descriptive anatomical comparison of the extensor forearm muscles of *Cebus libidinosus* [21], especially in comparison with new world monkeys. In the present study, we have expanded on those previous data by applying a non-parametric statistical test (Comparative Anatomy Index) [20] to compare the anatomy of the forearm flexor of muscles of *Cebus libidinosus* with those of other primates that use tools (humans and chimpanzees) and to baboons, which has not be reported to show this behavior. We also further expanded the findings of the previous report by applying the same statistical test to forearm extensor muscles.

Table 1 summarizes the similarities and differences across the superficial muscles forearm from *Cebus*, *Homo*, *Pan* and *Papio*.

**Figure 1:**
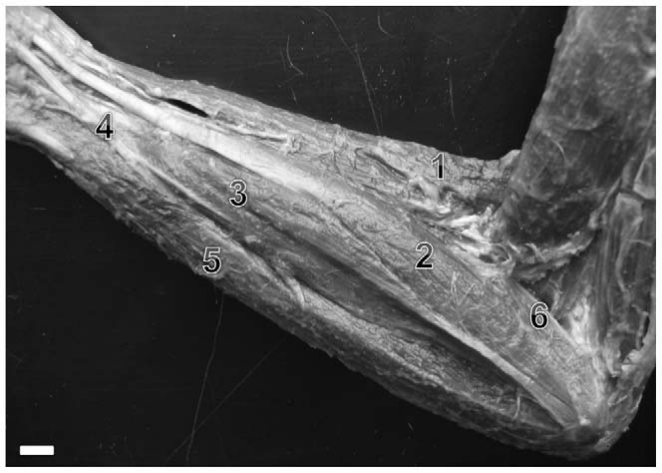
Photograph of the anterior aspect of forearm right of a *Cebus libidinosus (C.l.)*. 1).brachioradialis muscle, 2) flexor carpi radialis muscle, 3) flexor digitorum superficialis muscle, 4) palmaris longus muscle, 5) flexor carpi ulnaris muscle and, 6) pronator teres muscle. (bar = 1,2 cm).

According to Aversi-Ferreira and colleagues [22], the flexor carpi ulnaris muscle and palmaris longus muscle (Figure 1) are similar in all primates species analyzed here with regards to origin, insertion, innervation and vascularization. Flexor carpi radialis, flexor digitorum superficialis and pronator teres muscles, on the other hand, (Figure 1) are more similar between *Cebus* and *Papio*. The insertion of the flexor carpi ulnaris muscle on pisiform in *Cebus* is evident because this bone is well developed in this species.

In general, all superficial muscles in the *Cebus'* forearm present similarities to the other primates considered here. However, considering some specific details (shown in table 1), more similarities, based on the statistical analysis used in the present work, are found between superficial muscles in the *Cebus* and *Papio*.

Table 2 indicates the similarities and differences among the forearm deep muscles from *Cebus*, *Homo*, *Pan* and *Papio*, with regards to origin, insertion, innervation and vascularization.

**Table 2:** Comparative analysis of the flexor deep muscles forearm among *C.l.*, human (*Homo*), chimpanzee (*Pan*) and baboon (*Papio*).

Muscle	Features	*Cebus libidinosus*	*Homo*	*Pan*	*Papio*
**Pronator quadratus**	**Origin**	Internal portion antero-lateral of the distal third of the ulna	Innervated by median nerveSomewhat similar to *C.l.* **CAI = 0.375**	Highly similar to *C.l.* **CAI = 0.0**	Highly similar to *C.l.* **CAI = 0.0**
	**Insertion**	Border antero-medial of the distal third of the radius			
	**Innervation**	Ulnar nerve			
	**Vascularization**	Ulnar artery			
**Flexor digitorum profundus**	**Origin**	Proximal portion of the anterior surface of the ulna	Highly similar to *C.l.* **CAI = 0.0**	Not included tendon to index finger.Similar to *C.l.* **CAI = 0.0625**	Tendons from radial head to I, II and III fingers; and from ulnar head to III, IV and V fingers; associated with flexor digitorium superficial. To **CAI** purposes, it was considered inexistent.Dissimilar from *C.l.* **CAI = 1.00**
	**Insertion**	Base of the phalanges			
	**Innervation**	Ulnar nerve			
	**Vascularization**	Ulnar artery			
**Flexor pollicis longus**	**Origin**	Medial epicondyle of the antero-medial surface of the radius	Also originates from the adjacent part of the interosseous membrane.Similar to *C.l.* **CAI = 0.125**	Highly similar to *C.l.* **CAI = 0.0**	Attached to belly of the flexor digitorum profundus muscle.Somewhat similar to *C.l.* **CAI = 0.250**
	**Insertion**	Distal phalange of the thumb and a tendon to index finger			
	**Innervation**	Median nerve			
	**Vascularization**	Ulnar artery			
**Deep muscles**			Similar **GCAI = 0.167**	Similar **GCAI = 0.020**	Somewhat similar **GCAI = 0.417**

**Figure 2:**
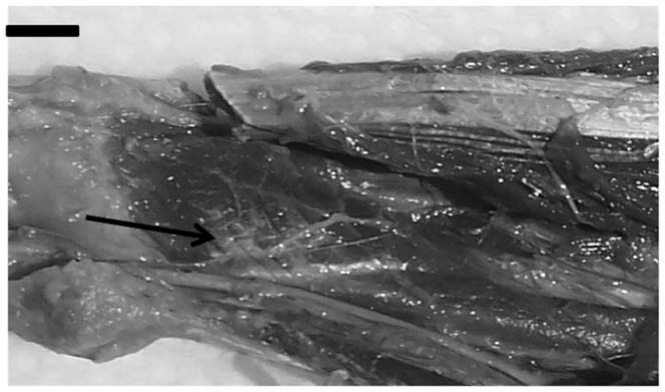
Photograph of the anterior aspect of forearm right of *C.l.* The arrow is indicating the pronator quadratus muscle. (bar = 0,5 cm).

Pronator quadratus muscle (Figure 2) is identical in all non-human primates considered. In humans, however this muscle is innervated by the median nerve [18], not the ulnar nerve as shown in non-human primates.

**Figure 3:**
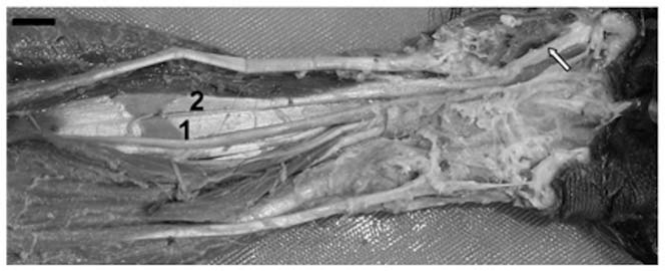
Photograph of the anterior aspect of forearm left of *C.l.* The arrow is indicating the tendon of flexor pollicis longus. 1) flexor digitorum profundus muscle and 2) flexor pollicis longus muscle. (bar = 0,7 cm).

The features observed in table 2 indicate that, in general, *Cebus* and *Pan* show great similarity regarding the flexor digitorum profundus and flexor pollicis longus muscles (CAI = 0.0625 and CAI = 0.0, respectively; also Figure 3), closely followed by Homo (CAI = 0.0 and CAI = 0.125, respectively). Interestingly, these muscles are more distinct in baboons, especially the digitorum profundus (CAI = 1.0), which was not the case for any superficial flexor muscles. The flexor digitorum profundus and flexor pollicis longus muscles are involved in finger (including thumb) movement. This anatomical evidence is consistent with the *Cebus'* manipulation skills.

The bellies of the flexor digitorum profundus and flexor pollicis longus muscles are clearly attached to each other, in contrast to humans where bellies are separated and individualized [18]. Indeed, these differences allow for the hand skills required by capuchins' arboreal habits, such as grabbing and holding [23].

**Figure 4:**
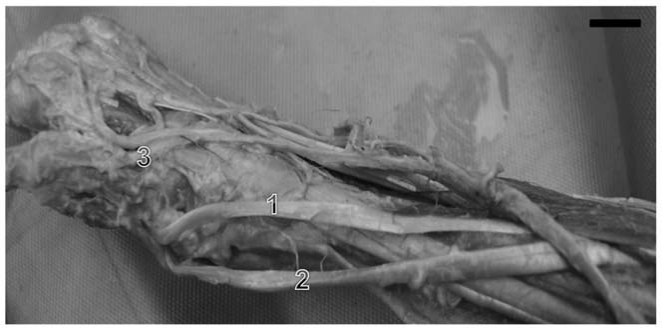
Photograph of the posterior aspect of right forearm of *C.l.* 1) tendon of the extensor pollicis brevis, 2) tendon of the abductor pollicis longus muscle, 3) tendon of the extensor pollicis longus muscle. (Bar = 1,2).

The descriptive analysis of the extensor muscles has been detailed in length elsewhere [21] (shown here in Figure 4). Here we provide a brief assessment of those findings under the light of CAI. In Table 3, we show the CAI and GCAI values for the extensor forearm muscles. High similarity (i.e. CAI = 0) between *Cebus*, *Pan* and *Papio* is evident in almost all extensor muscle, except for the deep dorsal sub-group. In this sub-group, the *Cebus'* abductor pollicis longus and extensor pollicis brevis show a greater similarity to modern humans and chimpanzees, respectively. It is important to note that the extensor pollicis brevis is not completely differentiated as a distinct muscle from the abductor pollicis longus in *Cebus* or in any other primate, except for humans and gibbons. The fleshy part, which constitutes this bundle, is deeply blended but it is differentiated into two separate tendons [21]. This configuration is highly similar to that of *Pan* and it is further differentiated in *Homo*, but not seen in *Papio*. Interestingly, the deep dorsal sub-group was pointed by [21] as the major point, in the forearm, which sets capuchins apart from the remaining new world monkeys.

**Table 3:** Comparative analyses of the extensor muscles forearm among *C.l.*, human (*Homo*), chimpanzee (*Pan*) and baboon (*Papio*). (Based on Aversi-Ferreira et al., 2010).

Muscle	Features	*Cebus libidinosus*	*Homo*	*Pan*	*Papio*
Radial Group					
Brachioradialis muscle	Origin	Latero-distal portion of the humerus on supracondylar ridge	Highly similar to C.l.CAI = 0.0	Highly similar to C.l.CAI = 0.0.	Highly similar to C.l.CAI = 0.0
	Insertion	Styloid process of radius			
	Innervation	Radial nerve			
	Vascularization	Radial artery			
Extensor carpi radialis longus	Origin	Latero-distal portion of the humerus on the supracondylar ridge	No attached bellies with others muscles.Somewhat similar to C.l.CAI = 0.22	Highly similar to C.l.CAI = 0.0	Highly similar to C.l.CAI = 0.0
	Insertion	Dorsal surface of the base of the second metacarpal on its radial side			
	Innervation	Radial nerve			
	Vascularization	Radial artery			
Extensor carpi radialis brevis	Origin	Lateral epicondyle of the humerus	Highly similar to C.l.CAI = 0.0	Highly similar to C.l.CAI = 0.0	Highly similar to C.l.CAI = 0.0
	Insertion	Dorsal surface of the base of the second metacarpal			
	Innervation	Radial nerve			
	Vascularization	Radial artery			
Supinator	Origin	Lateral epicondyle of the humerus	Highly similar to C.l.CAI = 0.0	Highly similar to C.l.CAI = 0.0	Highly similar to C.l.CAI = 0.0
	Insertion	Medium portion of the radious			
	Innervation	Radial nerve			
	Vascularization	Radial artery			
Superficial Dorsal Group					
Extensor digitorum communis	Origin	Lateral epicondyle of the humerus	Lesser variation regarding the distribution of tendons to fingers.Somewhat similar to C.l.CAI = 0.22	Highly similar to C.l.CAI = 0.0	Highly similar to C.l.CAI = 0.0
	Insertion	Dorsal aponeurosis in the second to fifth proximal phalanges			
	Innervation	Radial nerve			
	Vascularization	Radial artery			
Extensor digiti quinti proprius	Origin	Lateral epicondyle of the humerus	Only one insertion tendon to little finger.Somewhat similar to C.l.CAI = 0.22	Fleshy portion is well detached.Somewhat similar to C.l.CAI = 0.22	Highly similar to C.l.CAI = 0.0
	Insertion	Dorsum of the IV and V fingers			
	Innervation	Radial nerve			
	Vascularization	Radial artery			
Ulnar Group					
Extensor carpi ulnaris	Origin	Lateral epicondyle of the humerus	Highly similar to C.l.CAI = 0.0	Highly similar to C.l.CAI = 0.0	Highly similar to C.l.CAI = 0.0
	Insertion	Metacarpal of the little finger			
	Innervation	Radial nerve			
	Vascularization	Posterior interosseous artery			
Deep Dorsal Group					
Extensor pollicis longus	Origin	Posterior surface in the medium third of the ulna and interosseous membrane	Single insertion in distal phalange of the thumb.Somewhat similar to C.l.CAI = 0.250	Describe as derived from of a common extensor muscle primitive.Somewhat similar to C.l.CAI = 0.22	Similar to Pan.Somewhat similar to C.l.CAI = 0.22
	Insertion	Bases of the proximal and distal phalanges of the thumb			
	Innervation	posterior interosseous nerve			
	Vascularization	posterior interosseous artery			
Extensor indicis propius	Origin	Posterior surface of the ulna and interosseous membrane	Tendon isolated to index finger.Somewhat similar to C.l.CAI = 0.22	Highly similar to C.l.CAI = 0.0.	Highly similar to C.l.CAI = 0.0
	Insertion	Proximal phalanges of the II, III and IV fingers			
	Innervation	Posterior interosseous nerve			
	Vascularization	Posterior interosseous artery			
Abductor pollicis longus	Origin	Posterior surface of the ulna, radius and interosseous membrane	Highly similar to C.l.CAI = 0.0	Double insertion into the trapezoid and base of the first metacarpal.Somewhat similar to C.l.CAI = 0.250	Similar to Pan.Somewhat similar to C.l.CAI = 0.250
	Insertion	Base of the first metacarpal			
	Innervation	Posterior interosseous nerve			
	Vascularization	Posterior interosseous artery			
Extensor pollicis brevis	Origin	Proximal third of the radius and interosseous membrane	Single insertion in distal phalange of the thumb,Somewhat similar to C.l.CAI = 0.250	Highly similar to C.l.CAI = 0.0	Absent. Dissimilar from C.l.CAI = 1.0
	Insertion	articular capsule of the trapezoid-metarcapal I articulation and the base of this last bone			
	Innervation	posterior interosseous nerve			
	Vascularization	posterior interosseous artery			
Extensor group			Similar GCAI = 0.125	SimilarGCAI = 0.063	SimilarGCAI = 0.134

Since the majority of forearm muscles acts on the hand and fingers, the present study described the anatomy of the capuchin forearm muscles and compared them with those of humans, chimpanzees and baboons. The superficial group of flexor muscles was more similar to those of baboons (GCAI = 0.00) than chimpanzees and humans (GCAI = 0.20 and GCAI = 0.26, respectively). On the other hand, the deep flexor muscles were more similar to those of chimpanzees (GCAI = 0.02). They were even found more similar to those of humans (GCAI = 0.167) than those of baboons (GCAI = 0.417). The same pattern was found for extensor muscles, where capuchins were overall more similar to chimpanzees and humans (GCAI = 0.063 and GCAI = 0.125, respectively) than to baboons (GCAI = 0.134).

Baboons are mainly terrestrial monkeys with no reported use of tools either in captivity or in the wild [24]. Capuchins and chimpanzees are both arboreal and terrestrial, and even show an occasional bipedalism [25], [26]. Higher similarities in deep flexor muscles and extensor muscles between capuchin and chimpanzee, as opposed to baboon, suggest a possible link between lifestyle and forearm morphology. For instance, baboons do not show a clear separation among the extensor indicis propius, the extensor digiti quinti proprius and extensor pollicis brevis muscles, as seen in capuchin and chimpanzee. The only exception among extensor muscles is the extensor pollicis longus muscle. Also, the insertions of the extensor muscles in chimpanzees and capuchins are similar between both species but distinct from those in humans. They reflect the predominance of muscular strength over fine hand skills, which is associated with arboreal habits [21]. Nevertheless, Aversi-Ferreira et al., [27] noted that the capuchin shoulder and arm muscles, which aid in locomotion with thoracic members, are more similar to baboons than chimpanzees.

Another important factor regarding complex tool use is thumb opposability. Contrary to apes and macaques, capuchins present only lateral opposability [8]. This concept incorporates thumb prehensive grips observed in this genus. This finding was later corroborated by [21] which pointed to 3 thumb related movements that distance *Cebus* from NWM, namely: the extensor pollicis longus inserts in digit 1 only, abductor pollicis longus' anterior part is separated into 2 tendons, and extensor pollicis longus is not completely blended with extensor indicis. These features allow the uncoupling of the movements of the thumb from other digits. These are important differences that set capuchins away from closely related NWM. In the case of capuchin abductor pollicis longus, there is even higher similarity to humans than chimpanzees and baboons (CAI = 0.0; CAI = 0.250; and CAI = 0.250, respectively). The conjunct rotation that occurs at the capuchin carpo-metacarpal joint, which also allow this relative opposability, is more similar to OWM than NWM [28]. These findings confirm the evolutionary convergence of hand and forearm anatomy between capuchins and OWM, particularly chimpanzees and humans. They also support the high proximity in grasping and manipulative tasks among these species [9], [10], [11].

Finally, the use of fine, independent hand movement and thumb opposability for a wide variety of grasping and manipulation in capuchins is further supported by abundant corticospinal terminations [29]. These terminations are very dense at the ventral horn of cervical segments of the spine, from where motorneurons originate to innervate hand muscles. Rilling and Insel [30] also suggested that the increased number of sensorimotor fibers in *Cebus* brain may contribute to the wide variety of grasping strategies and manipulation skills. Capuchins also show high level of encephalization indices, in some cases, rivaling those of chimpanzees [30], [31], [32]. The highly developed cognitive skills shown by *Cebus* [33], [34] are also critical to solving tasks that requires tools.

Overall, capuchins' muscle and neural organization as well as behavioral habits and lifestyle point to an evolutionary convergence with chimpanzees, and even humans, despite a phylogenetic branching of around 30 million years ago [12]. The role phylogenetic constraints on the evolution of the forearm muscles of NWM cannot be underplayed: most of these muscles are remarkably similar across this very diverse group of primates [21]. The myological uncoupling of thumb movement and independent finger movements found capuchins, unique among NWM, is an important adaptive. The acquisition of such features may have allowed for the neurological and cognitive developments of tool use behavior.

In conclusion, the forearm anatomical data amassed from the present study as well as previous ones [21], [22] support the behavioral grasping and manipulation abilities observed in this genus. Forearm muscle shape and differentiation in capuchins is in keeping with capuchin's high encephalization indices and cognitive skills. These findings further corroborate the evolutionary convergence towards an adaptive behavior (tool use) between *Cebus* genus and apes.
